# Erythropoietin-Induced Hypertension: A Review of Pathogenesis, Treatment, and Role of Blood Viscosity

**DOI:** 10.7759/cureus.12804

**Published:** 2021-01-20

**Authors:** Simrandeep K Brar, Sumera Perveen, Muhammad Reza Chaudhry, Sarah AlBabtain, Sana Amreen, Safeera Khan

**Affiliations:** 1 Internal Medicine, California Institute of Behavioral Neurosciences & Psychology, Fairfield, USA; 2 Internal Medicine/Family Medicine, California Institute of Behavioral Neurosciences & Psychology, Fairfield, USA; 3 Family Medicine, Ibne Sena hospital Parco MCR, Multan, PAK; 4 Psychiatry, California Institute of Behavioral Neurosciences & Psychology, Fairfield, USA; 5 Department of Public Health and Preventive Medicine, St. George's University School of Medicine, St. George's, GRD; 6 Psychiatry and Behavioral Sciences, California Institute of Behavioral Neurosciences & Psychology, Fairfield, USA

**Keywords:** hypertension, erythropoietin, blood viscosity

## Abstract

Anemia is a common complication of certain chronic diseases and can be treated by stimulating hematopoietic cells to increase red blood cell count, and this action is achieved by recombinant human erythropoietin. In this review article, we have discussed about hypertension, which develops as a result of erythropoietin therapy. We have explored the pathogenesis of erythropoietin-induced hypertension and discussed some ways to prevent and treat this condition. Also, an attempt has been made to find out the role of blood viscosity in erythropoietin-induced hypertension. We conducted a comprehensive review of literature by collecting data from online databases like PubMed and Google Scholar. We mainly studied clinical trials that unraveled the mechanism of hypertension caused by erythropoietin. Hypertension is mainly caused due to enhanced vascular responsiveness to constrictors and impaired action of vasodilators. Role of blood viscosity in the pathogenesis of hypertension is doubtful due to the lack of consistency in the studies. Incidence of hypertension can be reduced by achieving slow correction of anemia and by switching to subcutaneous route of administration. Conventional anti-hypertensives have been found to be beneficial in the treatment. In some severe and persistent cases, temporary discontinuation of erythropoietin may be needed.

## Introduction and background

Erythropoietin is a cytokine secreted by kidneys, which promotes the formation of red blood cells. Erythropoietin and its derivatives have revolutionized the treatment of anemia due to diseases like chronic kidney disease (CKD), malignancy, and human immunodeficiency virus (HIV) infection [1]. Besides the correction of anemia, erythropoietin also has cardioprotective, renoprotective, and neuroprotective functions [2]. It has become a drug of abuse by athletes in order to increase their performance. Given its beneficial effects, erythropoietin is produced by recombinant deoxyribonucleic acid (DNA) technology, and this recombinant human erythropoietin (rhEPO) and its newer analogues [2] are now therapeutically used for the treatment of anemia.

However, it should be borne in mind that rhEPO does not come free from side effects. Hypertension, thrombosis, and stroke are the well-known complications of erythropoietin, especially when it is used to achieve high hemoglobin target levels [3]. In some cases, these complications can be life-threatening, [4] while in others, it can only be a temporary phenomenon of hemodynamic changes [5]. Thus, it is prudent to use the drug only when needed and to achieve hemoglobin levels that improve the quality of life of patients with anemia.

One common side effect of erythropoietin use is hypertension, which can occur in both healthy and diseased patients taking this drug. The incidence of hypertension with erythropoietin use is estimated to be around 10% to 15% [6]. It may develop anytime between two weeks and four months after the start of therapy [7]. Although different mechanisms have been postulated behind this, especially discussing vasoactive effects of erythropoietin on blood vessels, still much need to be known. Thus, this is a topic that merits further research. Various studies have been done discussing the role of nitric oxide, angiotensin, and intracellular calcium in the pathogenesis of hypertension [8-10]. There has been doubt around the association of increase in blood viscosity with increase in blood pressure. Newer studies have refuted this association [11]. This is another area of interest that needs to be discussed.

Erythropoietin can worsen cardiovascular functioning in patients with CKD. Uremic patients may have additional risk factors that put them at risk of hypertension with rhEPO use. For example, in CKD patients, levels of adrenomedullin have been found to be high, although its role in hypertension is uncertain [12]. A good number of clinical trials in both humans and animals have been done to explore additional mechanisms leading to hypertension in these patients.

In this review article, we have discussed in detail about the mechanism behind hypertension with erythropoietin use. Also, we have tried to find if any relationship exists between blood hematocrit changes and blood pressure. An attempt is made to find ways to mitigate hypertensive effects of erythropoietin, which can be done by slow infusion or by changing the route of administration.

## Review

Method

We used different keywords such as erythropoietin and hypertension alone and in various combinations to look for data on two databases, namely PubMed and Google Scholar. We screened articles by reviewing the title, abstract, and the full text. After thorough screening and removal of articles that did not meet our criteria, 29 articles remained relevant and were included in our study. We reviewed articles published in English language only. Clinical trials done on both humans and animals were reviewed by us. We reviewed full text studies as well as the studies in which only abstracts were accessible.

Result

Using the keywords, initially 56,590 results were generated. After removing duplicates and screening articles by reading the title, certain studies were removed to have 187 studies. Out of the 187 studies selected, we further removed 58 studies that seemed irrelevant to our topic to finally have 29 studies. These 29 studies mainly included clinical trials done both on humans and animals, observational studies and review articles.

Discussion

Pathogenesis of erythropoietin-induced hypertension

The possible mechanisms of hypertensinogenic effect of erythropoietin have been studied in animals as well as humans: both normal subjects, patients with anemia due to other causes and CKD patients. Cardiac output and systemic resistance increase after rhEpo therapy, which is one mechanism by which erythropoietin exerts hypertensinogenic effects [5]. Increase in systemic resistance leading to hypertension is due to vasoconstrictor effects of erythropoietin on small resistance vessels as studied by Heidenreich et al. in isolated renal and mesenteric vasculature of male rats [13]. Also, erythropoietin has been found to impair acetylcholine-mediated vasodilator response [14]. Impaired vasodilation leads to unopposed constriction of the blood vessels and hence hypertension.

A study by Bode-Böger et al. on isolated rabbit arteries revealed that rhEPO increases the synthesis of endothelin-1 and constrictor prostanoids, thus increasing vascular responsiveness to noradrenaline [15]. Further, hypertension may develop due to altered balance between the vasodilator and vasoconstrictor factors. This effect was studied by Shimada et al. who found raised endothelin-1 to nitric oxide ratio in vascular endothelial cells in hypertensive hemodialysis patients [8]. Calcium levels inside the smooth muscle cells of blood vessels also have a role to play. As proposed by Marrero et al., erythropoietin increases calcium influx in smooth muscle cells, thus causing vasoconstriction [10].

Additionally erythropoietin exerts antinatriuretic effect, and by altering sodium excretion, it can lead to hypertension [9]. This antinatriuretic effect of erythropoietin is mediated by angiotensin II [9]. Kuriyama et al. found that the individuals that carry a particular allele at angiotensinogen gene are more susceptible to hypertension induced by erythropoietin treatment [16]. Thus, it indicates that angiotensin is an important factor mediating the hypertensive effect of erythropoietin and polymorphism of the angiotensinogen gene has a bearing on the development of hypertension.

**Figure 1:**
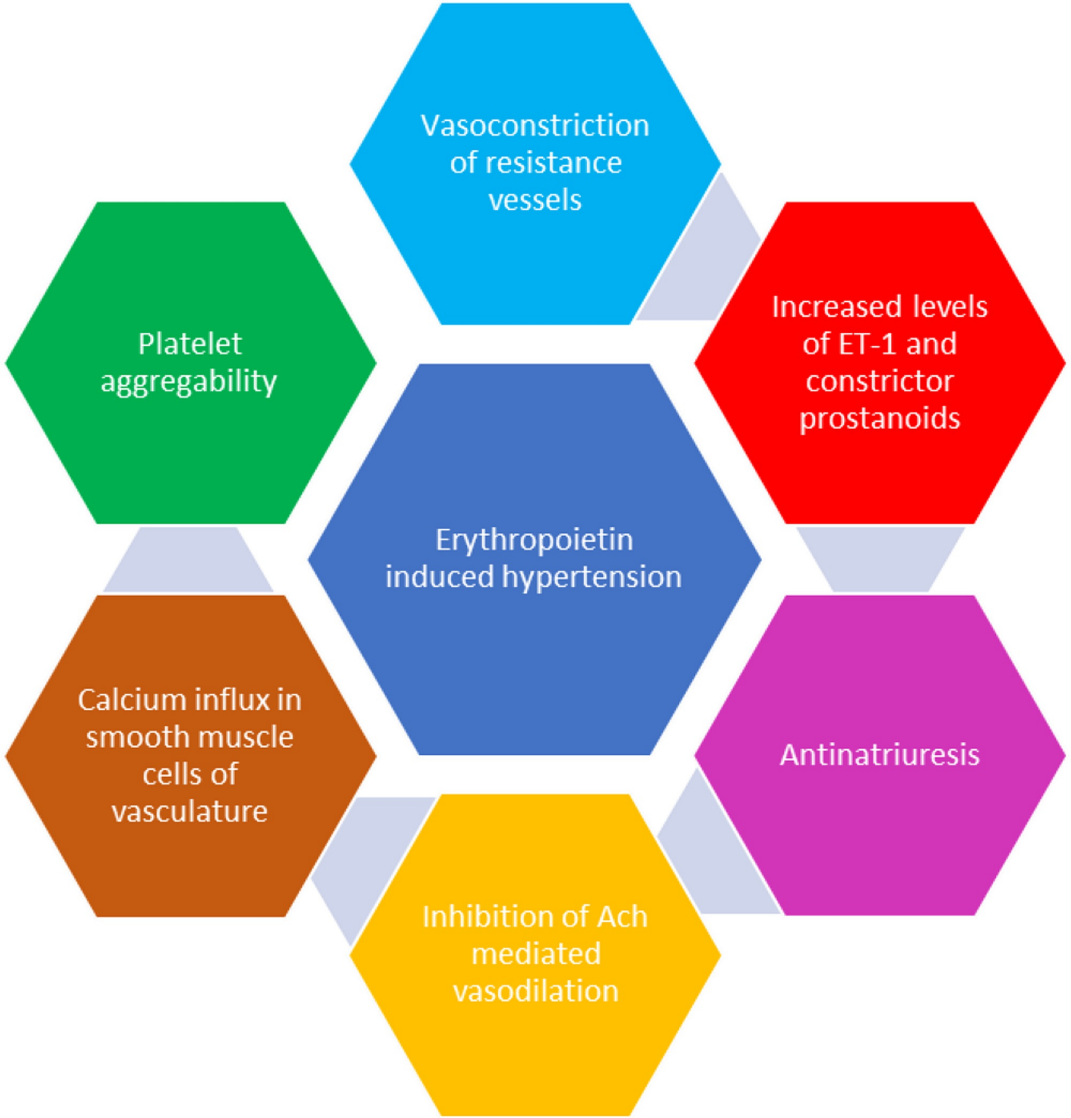
Mechanism of erythropoietin-induced hypertension ET-1, Endothelin 1; Ach, Acetylcholine.

A retrospective observational study done by Caravaca et al. on uremic patients revealed that antiplatelet therapy may prevent the incidence of hypertension caused by rhEPO therapy [17]. This indicates the role of platelet aggregability in the occurrence of hypertension [17]. Some of these mechanisms postulated play more important role in the development of hypertension in the patients with CKD than in the patients with anemia due to other causes. Table 1 mentions about the studies conducted to find out the mechanism of hypertension after erythropoietin therapy. Figure 1 summarizes the mechanism of erythropoietin-induced hypertension.

**Table 1:** Studies done to elucidate the mechanism behind erythropoietin-induced hypertension EPO, Erythropoietin; ECF, extracellular fluid; rhEPO, recombinant human erythropoietin; BP, blood pressure; NO, nitric oxide; ET-1, endothelin-1; Ach, acetylcholine; IC, intracellular; SBP, systolic blood pressure; DBP, diastolic blood pressure; MAP, mean arterial pressure.

Author	Intervention studied	Number of participants	Type of study	Result	Conclusion
Krapf et al. [18]	Erythropoietin’s effect on BP	-	Review	EPO causes hypertension in normal subjects, dialysis, and predialysis patients.	Hypertensinogenic effect is underreported and not fully studied.
Kuriyama et al. [16]	EPO and angiotensinogen gene	51	Clinical trial	Individuals homozygous for particular allele are at greater risk of EPO-induced hypertension.	Angiostensinogen gene polymorphism is associated with EPO-induced hypertension.
Caravaca et al. [17]	Antiplatelet therapy and EPO	91	Observational study	Low incidence of hypertension in patients taking antiplatelet therapy.	Antiplatelet therapy may prevent rhEPO-induced hypertension.
Kuriyama et al. [12]	Adrenomedullin levels in EPO-induced hypertension	54	Clinical trial	EPO did not alter plasma concentration of adrenomedullin.	Role of adrenomedullin in EPO-induced hypertension is uncertain.
Migliori et al. [19]	EPO’s effect on nitric oxide (NO) levels		Clinical trial	Urinary excretion of NO metabolites was higher in treatment group.	NO activity was enhanced in rhEPO-induced hypertension.
Brier et al. [9]	EPO’s effect on sodium excretion		Clinical trial	Antinatriuretic effect of EPO is mediated by angiotensin II.	Antinatriuresis can be the cause of EPO-induced hypertension.
Noshad [20]	EPO’s differential effect on BP increase	80	Clinical trial	EPO injection increases SBP, DBP, and MAP in hemodialysis patients and only SBP in predialysis patients.	BP increase with EPO is more pronounced in hemodialysis patients.
Kilar et al. [14]	EPO’s effect on Ach-mediated vasodilation	6	Clinical trial	EPO activated β receptors in mouse mesenteric arterioles.	EPO impairs Ach-mediated vasodilation.
Bode-Böger et al. [15]	rhEPO’s effect on endothelin-1 and prostanoid levels		Clinical trial	rhEPO increases synthesis of endothelin-1 and constrictor prostanoids.	rhEPO enhances vascular responsiveness to noradrenaline.
Marrero et el. [10]	EPO’s effect on calcium channels		Clinical trial	EPO increases calcium influx in vascular smooth muscle cells.	Vasoconstrictive effect of EPO leads to hypertension.
Heidenreich et al. [13]	Effect of rhEPO on resistance vessels	30	Clinical trial	rhEPO causes smooth muscle contraction of small resistance vessels.	Vasoactive properties of rhEPO contribute to hypertension.
Lebel et al. [21]	Effect of rhEPO on hormones	32	Clinical trial	rhEPO increases aldosterone levels.	Reducing ECF volume can decrease hypertensive effect.
Okura et al. [22]	Effect on EPO on IC sodium levels	11	Clinical trial	EPO therapy after three months leads to an increase in the IC sodium levels.	IC sodium accumulation may lead to BP elevation.
Shimada et al. [8]	Effect of rhEPO on ET-1/NO ratio	15	Clinical trial	ET-1/NO ratio was high in hypertensive hemodialysis patients given rhEPO.	Increase in ET-1/NO ratio can lead to rhEPO-induced hypertension.

Role of blood viscosity

There is no clear consensus on the role of blood viscosity in erythropoietin-induced hypertension. It is believed that increase in hematocrit and blood viscosity after treatment with recombinant human erythropoietin alters vascular responsiveness and increases vascular resistance, which leads to hypertension [23]. However, some of the studies conducted later revealed that there is no such association between hypertensive response and rise in hematocrit [24]. A study done by Cirillo et al. on Gubbio population revealed that the prevalence of hypertension was two times higher in persons whose hematocrit levels were high by 10 units [25]. Among the 2809 participants recruited, they found that independent of other confounders, the hypertensive group had a higher hematocrit than the non-hypertensive ones [25]. Also, it was found by Fishbane et al. that high target hemoglobin level achieved is associated with increased risk of adverse effects such as hypertension and other cardiovascular effects [3]. Thus, optimal hemoglobin levels need to be achieved rather than too high levels in order to improve the quality of life of patients with anemia without subjecting them to adverse effects of erythropoietin.

However, Samtleben et al. were the first to show that the rise in blood pressure did not correlate with the rise in hematocrit in all patients, thus suggesting that the pressor mechanisms and not blood viscosity played a role [15,24]. Blood pressure decreases after transfusion despite rise in hematocrit and blood viscosity [26]. This observation further questions the role of blood viscosity in erythropoietin-induced hypertension. A study was done by Canaud et al. in which they evaluated blood viscosity and blood pressure changes in 13 patients undergoing hemodialysis who received rhEPO for one year or longer. Hypertension was seen in only three patients receiving rhEPO, whereas increase in blood viscosity was seen in all [11]. Noshad conducted a study in which a rise in blood pressure was detected in end-stage renal disease patients who received erythropoietin therapy. It was noted that the hypertensive effect of erythropoietin was evident much before its hyperviscosity effect. Thus, the results showed that hypertension induced by erythropoietin was independent of hyperviscosity [20].

The literature reviewed showed inconsistent findings with some studies supporting the claim that rise in blood viscosity leads to hypertension after erythropoietin therapy, while other studies gave a contradictory view. Due to the lack of clear evidence and no consensus over the subject, no definite idea can be derived. Being a debatable subject, this area merits further investigation in order to reach a definite conclusion.

Prevention and treatment

Since erythropoietin therapy is well known to cause hypertension, the patients receiving erythropoietin must be monitored for their blood pressure changes. In patients on dialysis, by optimizing dialysis treatment and by paying close attention to volume changes, the occurrence of high blood pressure can be reduced [27]. High-dose erythropoietin therapy intended to achieve high hemoglobin target levels should be avoided as it increases the risk of blood pressure changes [3]. Optimal levels of hemoglobin should be achieved, thus minimizing the side effects. Hypertensive complications can be reduced by slow correction of anemia [5].

Hypertension that developed as a result of erythropoietin therapy can be controlled by conventional antihypertensive therapy, but if it persists, then reduction in the dose of erythropoietin or its temporary discontinuation may be needed [23]. Changing the route of administration from intravenous to subcutaneous has been found to be beneficial in controlling blood pressure increase. Navarro et al. conducted a study in 13 hemodialysis patients who were hypertensive after more than one year of intravenous rhEPO therapy. On switching to subcutaneous route of administration of rhEPO, not only the blood pressure control improved in hypertensive patients but also their dose requirement of rhEPO decreased [28]. Platelet aggregability plays an important role in the development of rhEPO-induced hypertension. Hence, antiplatelet drugs may prevent the development of hypertension in rhEPO therapy [17].

Limitations

We could not derive a definite conclusion regarding the role of blood viscosity in erythropoietin-induced hypertension as the literature reviewed had contradicting results. Hence, due to the lack of consistency, no clear idea could be obtained. Also, certain studies being in languages other than English and some being inaccessible could not be reviewed by us.

## Conclusions

Erythropoietin has revolutionized the treatment of anemia due to chronic diseases and malignancy. It is widely used in the form of recombinant human erythropoietin due to its hematopoietic activity. Hypertension is a well-known side effect of rhEPO that can develop due to increase in the cardiac output, systemic resistance, and increased level of endothelin-1 and constrictor prostanoids. Besides that, platelet aggregability, antinatriuresis, and impaired vasodilatory action of acetylcholine play an important role in the pathogenesis of hypertension after rhEPO therapy. We tried to explore the role of blood viscosity in the development of hypertension but could not get a definite answer due to various studies conducted giving contradicting results. More research is needed in this subject. However, we concluded that to minimize the risk of hypertension and other adverse effects, anemia should be corrected slowly and not very high target hematocrit levels should be tried to achieve. Also, regular blood pressure monitoring is needed in patients receiving erythropoietin therapy as both normotensive and previously hypertensive patients can develop hypertension post-therapy.
